# The STR/ort mouse model of spontaneous osteoarthritis – an update

**DOI:** 10.1016/j.joca.2016.12.014

**Published:** 2017-06

**Authors:** K.A. Staines, B. Poulet, D.N. Wentworth, A.A. Pitsillides

**Affiliations:** † Roslin Institute and R(D)SVS, The University of Edinburgh, Easter Bush, Midlothian, EH25 9RG, UK; ‡ School of Applied Sciences, Edinburgh Napier University, Sighthill Campus, Edinburgh, EH11 4BN, UK; § Institute of Ageing and Chronic Diseases, Musculoskeletal Biology 1, University of Liverpool, Room 286, Second Floor, William Henry Duncan Building, 6 West Derby Street, Liverpool, L7 8TX, UK; ‖ The Royal Veterinary College, Royal College Street, London, NW1 0TU, UK

**Keywords:** STR/ort, Osteoarthritis, Articular cartilage, Subchondral bone

## Abstract

Osteoarthritis is a degenerative joint disease and a world-wide healthcare burden. Characterized by cartilage degradation, subchondral bone thickening and osteophyte formation, osteoarthritis inflicts much pain and suffering, for which there are currently no disease-modifying treatments available. Mouse models of osteoarthritis are proving critical in advancing our understanding of the underpinning molecular mechanisms. The STR/ort mouse is a well-recognized model which develops a natural form of osteoarthritis very similar to the human disease. In this Review we discuss the use of the STR/ort mouse in understanding this multifactorial disease with an emphasis on recent advances in its genetics and its bone, endochondral and immune phenotypes.

## Introduction

Animal models are a vital tool for the study of osteoarthritis (OA). In particular they provide scope to examine the early aetiological processes where equivalent human samples are difficult to obtain, and they remain necessary in developing and testing new treatments^1^. Animal OA models consist broadly of those requiring invasive manipulation, such as surgical joint destabilization by ligament transection or meniscectomy, those destabilizing the joint without surgical manipulation such as collagenase-induced instability, those exploiting non-surgical application of mechanical trauma, or those in which OA develops naturally^2^, ^3^, ^4^. Surgical models are representative chiefly of trauma-induced secondary human OA, whilst natural models offer an opportunity to investigate OA without known aetiology, akin to primary OA^5^. All of these OA models principally use the mouse; well-used mainly due to the ease with which its genome can be manipulated and because of its robust breeding capacities, easy husbandry and price of upkeep. In addition, its relatively short life-span allows for the examination of OA progression, from initiation to late degenerative stages, to be undertaken in a compressed timescale.

Numerous mouse strains develop OA with advancing age, offering proof that genetic predisposition or susceptibility is an important factor. They, nonetheless, develop OA with differing incidences^6^; high incidence and severity was identified in the STR/1N mouse, from which the inbred STR/ort strain is directly derived. The STR/ort mouse is a well-recognized model of spontaneous OA and, to date, has featured in over 80 studies. STR/ort mice develop OA spontaneously early in life and show many human OA characteristics, including proteoglycan (PG) loss, articular cartilage (AC) fibrillation, active extracellular matrix (ECM) degradation, osteophyte formation and subchondral sclerosis. Herein, we will revisit (see Watanbe *et al.*, 2011 and Mason *et al.*, 2001^7^, ^8^) past and new data from STR/ort mice with view to revealing how they inform our understanding of early aetiology, pathophysiology and potential treatment of OA.

## Origins of the STR/ort mouse

The STR/1N strain was first isolated by Strong (1951) during an extensive selective-breeding programme designed to identify traits for resistance to tumour induction at the site of injected carcinogens^9^. Tandem crosses between CBA, N, J and K strains generated a new NH strain that was treated for multiple generations with the carcinogen, 20-methylcolanthrene creating the NHO strain. Further selection using another carcinogen (4-methylcholantrene) ended with a piebald mutation and serendipitous generation of the STR/1N strain^7^, which exhibited obesity and spontaneous OA at a young age^10^. After some breeding without brother-sister pairing and arrival at the Institute of Orthopaedics, Stanmore (UK), the strain was renamed STR/ort, as it is now commonly known. The CBA mouse is the only remaining parental strain available today and it's lack of overt OA makes it effective as a control^11^.

## STR/ort mouse OA phenotype

STR/ort OA susceptibility genetics are uncertain and their phenotype is better characterised. STR/ort mice develop OA in knee, ankle, elbow and temporomandibular joints^12^, ^13^, ^14^, ^15^. The first studies by Walton described a greater incidence of OA knee pathology in male than in female STR/ort mice; a sexual dimorphism in this model which is the opposite to that in the human disease^16^, ^17^. In male mice, Walton reported steadily increasing OA incidence and severity from 18 weeks of age. We have shown, by toluidine blue staining followed by the internationally-recognized OARSI grading system^18^, that the OA in the STR/ort mouse invariably predominates on the medial tibial plateau at the cruciate ligament insertion, is followed by AC clefting/fibrillation extending centrally and then later to medial femoral condyles and is accompanied by osteophyte development and by subchondral bone sclerosis which, with extensive AC loss across both condyles, later becomes exposed. Chondrogenesis and ossification in collateral/cruciate ligaments, and meniscal hyperplasia, ossification and eburnation is seen in severely affected joints. This is consistent with greater chondrocyte proliferation, synovium hyperplasia and cluster formation in menisci of STR/1N mice^19^ described as tentative evidence for reparative processes. It fails to match, however, with prominent synovial inflammatory infiltrates seen in STR/ort mice by some. Our recent work examining whether gait changes are a meaningful measure of STR/ort OA severity showed that age-related modifications in paw area precede OA onset and may therefore be useful for longitudinal monitoring of OA development in these mice^20^.

Only a few studies have explored OA-associated pain in this model. Increased basal and evoked prostaglandin E2 release has been observed in knee preparations from 18-week-old STR/1N mice, which may enhance nociceptor sensitivity and chronic OA pain^21^. However, we have found that male STR/ort mice do not exhibit any pain-associated behaviours with OA development, even when treated with the opioid antagonist naloxone^20^. They did however exhibit normal pain behaviours in response to complete Freund's adjuvant-induced arthritis, suggestive that these mice are not inherently insensitive to joint pain^20^. Despite this, the precise nature of OA pain in STR/ort mice is unresolved.

## Mechanical aetiology of OA in the STR/ort mouse

Several lines of evidence suggest that OA development in STR/ort mice involves a mechanical contribution. Indeed, Walton (1977) showed a close relationship between AC lesions and medial patella dislocation, medial collateral ligament calcification/ossification and lateral subluxation of the femur^16^ and that surgical patella fixation decreased OA, whereas patella dislocation in CBA mice induced OA^22^. Patella dislocation has also been linked to abnormal tibial internal torsion in STR/ort (STR/OrtCrlj) mice and to advanced age, leading Naruse *et al.* (2009) to propose this as the cause of STR/ort OA^23^. However, Das-Gupta *et al.*, reported an incidence of patella dislocation in only 22% of STR/ort mice, reinforcing this with radiological studies showing not all mice with OA had displaced patellae, demonstrating that this cannot be a primary event^24^. We find that patella dislocation correlates with severe OA, but can be absent even in some STR/ort mice with severe AC degeneration (unpublished data). Other studies have indicated that this medial patellar luxation in STR/1N mice is likely due to medial tibia AC degeneration, pronounced instability and varus knee joint deformity, which contrasts with the valgus characterising knee OA in C57/Bl6 mice^25^, ^26^. Mechanical changes induced by patella dislocation could nonetheless be an important contributor and aggravator of OA development in STR/ort mice.

Development of ankle OA in STR/ort mice has similarly been linked with calcaneal dislocation, with elevation progressively more pronounced in ageing mice where it eventually became parallel to the distal tibia (unpublished). The cause for this ankle deformity is unknown, but suggests a possible defect in maintaining joint stability with spontaneous subluxation and later severe disruption of navicular and tarsal bones in male STR/ort mice^15^. Together, these studies suggest a widespread instability phenotype that disrupts joint mechanics to promote OA. This was however deemed unlikely by scoring of multiple STR/ort mouse joints which found that patellar and calcaneal displacements rarely occurred in the same limb, suggesting they were likely independent events^12^.

The anterior cruciate ligaments of STR/ort mice also exhibit lower ultimate strength, increased collagen metabolism and matrix metalloproteinase (MMP) activity compared to CBA mice at 20–30 weeks^27^, ^28^. This suggests that STR/ort mice have inherently weaker ligaments, which could facilitate patella dislocation and joint instability. Changes in ligaments and menisci in STR/ort OA joints, with chondrogenesis and ossification, are also seen in surgical OA models, supporting their mechanical aetiology. These changes would, in turn, modify the mechanical properties of the ligaments and cause further joint damage.

We have recently explored the importance of mechanical loading in lesion induction and pathological OA progression in STR/ort mice using a non-invasive knee joint trauma model^29^. We found that AC in STR/ort mice is relatively resistant to mechanical trauma–it can bear greater applied loads without failure–which is associated with thicker AC at all ages relative to CBA mice^29^. These data suggest that STR/ort mouse OA susceptibility is unlikely due to enhanced vulnerability of AC to mechanical lesion induction. We did, however, find that repetitive mechanical loading over a two week-long period promoted progression of spontaneously-occurring AC lesions in the medial tibia, suggesting that mechanical disturbances may nevertheless accelerate OA progression in these mice^29^. This merges well with human studies showing that mechanical loading of joints is likely a major determinant of both OA onset and progression and further highlights the attractiveness of the STR/ort mouse as a model for exploring interplay with mechanical factors in OA development.

## Genetic studies in the STR/ort mouse

Numerous genetic and microarray analyses have been performed in STR/ort mice. Studies by Jaeger *et al.* (2008) confirmed Mendelian OA inheritance and concluded that its polygenicity means that the allelic subset involved in OA predisposition unlikely reaches significance in any single-Quantitative Trait Loci (QTL) analysis^30^. Genotyping of male F2 (STR/ort × C57BL/6) using 96 microsatellite markers and phenotyping by weight, serum COMP biomarker levels and knee OA revealed three weight-, one serum COMP- and one OA-associated QTL on chromosome 8^30^. Backcrossing F1 STR/ort male to C57BL/6N females and linkage by microsatellite markers, again showed polygenicity with a QTL for OA instead mapped to a 20 centimorgan region, proximal to chromosome 4's centromere (another linked to OA onset in C57BL/6N mice on chromosome 5 was identified)^31^; together these data might simply support the existence of multiple murine OA loci.

Revisiting chromosome 8 and fine mapping of the OA-QTL revealed Wnt-related genes associated with altered chondrogenesis, including *dickkopf 4* (*Dkk4*), *secreted frizzled related protein 1* (*Sfrp1*) and *fibroblast growth factor 1* (*Fgfr1*), with 23 polymorphic changes in the *Sfrp1* gene identified in STR/ort in comparison to C57BL/6 mice^32^, suggesting that reduced Sfrp1 expression not only increases Wnt/β-catenin signalling early in life but also renders the AC prone to premature OA^32^. This is similar to various genome-wide expression profiling studies in human OA which have also identified members of the Wnt/β-catenin signalling pathway as candidate genes associated with OA.^33^, ^34^

Our recent studies also support an epigenetic contribution to STR/ort OA (unpublished). Careful joint OA scoring in individually-tracked male mice at 26 weeks of age found an important maternal influence, with a significant correlation between OA severity and maternal litter parity, and to a lesser extent with maternal age. Interestingly, no correlations were found with litter size nor with the time between litters, which suggests an important maternal influence during embryo development that underpins OA severity in STR/ort mice.

## Articular cartilage phenotype of STR/ort mice

### Matrix remodelling

STR/ort mouse AC undergoes structural demise similar to human OA. Morphologically, STR/ort mouse AC is thicker than in CBA, and whilst STR/ort chondrocytes express a normal spectrum of PGs and collagens, there are early changes in AC matrix integrity and chondrocyte phenotype and function^35^, ^36^, ^37^. These include a subtle, yet progressive decay in PG orientation prior to any decline in quantity, which was proposed to reflect the increased free water-content characteristic of human OA^38^. In addition, STR/ort mouse AC catabolic and anabolic gene expression profiles closely resembled those seen in other mouse OA models and in human OA^11^, consistent with increased MMP expression and activity^37^, ^39^, ^40^. The importance of MMPs in AC degradation in STR/ort mice has been tested by administration of Ro 32-3555, an orally active collagenase selective inhibitor which protected against OA development (Table I)^41^. In addition, intra-articular injections of CRB0017 (anti-ADAMTS5 antibody) dose-dependently slowed OA progression in STR/ort mice ageing from 5 to 8 months (Table I)^42^. The likelihood that the STR/ort mouse model will help identify new preventative, protective and curative avenues targeting AC in OA joints is therefore well supported.

**Table I:** A summary table of some therapeutics tested in the STR/ort mouse model and their outcomes

Agent	Action	Outcome	Ref.
Naloxone	Opiod antagonist	No signs of pain-associated behaviours	20
Ro 32-3555	Collagenase inhibitor	Reduction in joint space narrowing, osteophyte formation and protection against AC degradation and subchondral bone sclerosis	41
CRB0017	Anti-ADAMTS5 antibody	Dose-dependently slowed OA progression in STR/ort mice ageing from 5 to 8 months	42
Vitamins E, C, A, B6, B2, and selenium	Dietary supplementation	Lower OA incidence	65

Subtle changes in STR/ort AC matrix composition, and in glycosaminoglycan PG content in particular, is observed in STR/ort male mice in comparison to age-matched CBA mice^36^, ^43^. Indeed, chondroitin sulphate content, predominantly C6S, is elevated in STR/ort mice at 8–19 weeks (before OA onset), decreases at 24–26 weeks of age, before increasing again thereafter (after OA onset)^36^. These changes in AC composition may therefore impact AC function prior to OA onset and this highlights potential targets for therapeutic intervention.

### Chondrocyte phenotype

Alternative approaches to redress the lack of therapeutics in OA are also now emerging. Evidence suggests that the normally ‘stable’ AC chondrocyte adopts a more ‘transient’ phenotype similar to growth plate chondrocytes in OA^35^, ^44^, ^45^, ^46^. This phenotype switching also occurs in STR/ort mice with the hypertophic marker *Col10a1* mRNA significantly increasing in STR/ort AC compared to non-OA AC^11^, ^35^, ^47^. Consistent with this, Col10a1 immunolabelling has been observed throughout AC of STR/ort mice before histological OA is detected^35^.

TUNEL-positive chondrocytes are observed around OA lesions in STR/ort AC, indicating apoptosis and chondrocyte transiency, correlating with OA progression^48^. The lack of any changes in the ratio of Bax:Bcl-2 in STR/ort AC^48^ indicates that this apoptosis is perhaps attributable instead to increased chondrocyte adenosine production^49^. Aberrant control of upstream regulators of apoptosis have also been found in STR/ort mouse AC; including prohibitin-1, a protein which restricts generation of reactive oxygen species (ROS), mitochondrial disorganization, abnormal cristae morphology and increased sensitivity towards stimuli-elicited apoptosis^50^. In both STR/ort, and human AC, accumulation of prohibitin-1 along with Pitx1 repression was detected in OA chondrocyte nuclei, consistent with elevated apoptosis.

It appears that STR/ort mouse AC chondrocytes also have an altered metabolic phenotype, with those in OA–prone regions having low lactate and succinate dehydrogenase activities prior to OA onset^51^, ^52^. This aberrant metabolic phenotype is also evident in lower glucose 6-phosphate dehydrogenase activity and different monoamine oxidase localisation specifically in AC regions where OA develops^53^, ^54^, ^55^; the latter exhibiting potential for therapeutic targeting in STR/ort mice.

### Cell signalling pathways

Discovery of the molecular determinants of OA in STR/ort mice will undoubtedly shed light upon OA aetiopathogenesis in other species and as such, have been well investigated. Our transcriptional profiling of STR/ort AC at various ages revealed differential regulation of many signalling pathways^11^, including an underexplored pathway relating to genes normally associated with the contractile machinery of muscle cells; expression of this gene subset is high in both young STR/ort and CBA mice, but remains high in OA STR/ort when a significant decrease is seen in healthy CBA aged samples^11^.

Major pathways such as those provoked by the transforming growth factor (TGF) β superfamily have already been investigated. Expression of TGF-β1 was indeed elevated during OA development in STR/ort compared to age-matched CBA mice^56^. Further, AC chondrocyte TGF-β3 and SMAD-2P protein expression decreased in STR/ort mice with advancing OA severity, except in areas of osteophyte formation where elevated levels persisted^57^; blocking studies suggest that TGF-β3 is involved in early and bone morphogenetic proteins (BMPs) in late osteophytogenesis^57^, ^58^.

Effective regulation of the Wnt pathway is proving critical in OA joint pathology^59^ and levels of sFRP1, the Wnt inhibitor, are reduced in AC chondrocytes of young STR/ort mice^32^. We have reported a role for another Wnt inhibitor, sclerostin. This shows marked enrichment at the osteochondral interface in the relatively unaffected lateral tibia but its expression was severely disrupted in medial tibial regions showing AC loss and subchondral bone thickening^35^. Similar differential expression patterns of matrix extracellular phosphoglycoprotein (MEPE), an inhibitor of cartilage matrix mineralisation^60^ and downstream sclerostin target^61^, were also observed in STR/ort mice, implicating a novel mechanism by which sclerostin, and hence Wnt signalling functions in OA^35^, ^59^. Hypoxia inducible factor 1 (HIF-1α) also plays a major role in joint homeostasis and its inhibition rapidly provokes OA development in Balb/C mice. Intriguingly, HIF-1α stabilisation failed to prevent OA in STR/ort mice^62^ which further supports use of STR/ort mice in discerning whether identical pathological pathways are common to all forms of OA.

### Oxidative stress

Oxidative stress has been shown to contribute to OA progression. In STR/ort mice, oxidative stress (malondialdehyde) and the collagen type II degradation (CTX-II) biomarker levels are both higher than in CBA mice prior to OA onset, suggesting that oxidative stress is linked to AC type II collagen degradation^63^. Even before OA onset, young STR/ort mice show decreased levels of extracellular superoxide dismutase, the major scavenger of extracellular ROS in AC, and elevated nitrotyrosine formation at all ages, suggesting that inadequate control of ROS plays a pathophysiological role in OA^64^. This role is supported by markedly lower OA incidence in STR/1N mice following dietary supplementation (Table I)^65^. More recently, apurinic/apyrimidinic endonuclease 2 (Apex 2) was also claimed to play a critical role in DNA repair caused by oxidative damage in STR/ort (STR/OrtCrlj) joints^66^.

## Bone phenotype of the STR/ort mouse

Subchondral bone thickening (sclerosis) in OA joints, although often considered secondary, is nonetheless one of the earliest detectable changes and we have observed sclerosis in STR/ort joints with OA onset and development^35^. This agrees with decreased osteoclastogenesis (85Sr incorporation), increased bone apposition that is spatially associated with AC lesions in early STR/ort mouse OA (polychrome sequential bone labelling) and with early MRI in STR/ort mice where changes in patellar tendon and local sclerosis were identified^67^, ^68^. They are also consistent with a recent comprehensive multimodal micro-computed tomography study which determined compartment-, age- and site-specific changes in subchondral bone in STR/ort mice evoking temporal changes that lead to an altered architecture contributing to their OA phenotype^69^.

STR/ort mice also have a generalised high bone mass phenotype in cortical and trabecular compartments too (vs C57Bl/6), associated with elevated osteoblast numbers and activity, and impaired osteoclast function^70^. Indeed, changes in bone remodelling have already been implicated in the early stages of STR/ort mouse OA, where raised urinary CTX-II levels were apparent in an OA subgroup of STR/ort mice (vs non-OA subgroup^71^). Consistent with an inherent bone phenotype, we have recently reported that young (6-week) STR/ort have increased cortical and trabecular parameters in comparison to age-matched CBA mice^35^. Surprisingly, this difference is noted significantly earlier and is more marked in female STR/ort mice, with an almost complete bone marrow compression and extramedullary haematopoiesis observed by 9 months^70^. This raises an interesting paradox regarding sexual dimorphism in this strain, where females show–on one hand–higher bone mass and protection from reproducible AC degeneration and where–on the other–male OA appears not to be influenced by hormone status^37^, ^72^. It therefore seems unlikely that high bone mass alone is sufficient to accelerate OA onset. Sexually dimorphic OA development might instead be due to architectural bone differences. Thus, early internal tibial torsion and lower cancellous bone mineral density evident in males may explain the differential incidence of OA in this STR/ort strain^73^.

Clues to these changing osteochondral relationships in STR/ort mice might be evident in the endochondral ossification required for long bone growth. We recently observed accelerated growth dynamics in comparison to CBA mice with STR/ort mice exhibiting (1) an acceleration in body weight gain and tibia length at sexual maturity (2) Col10a1 and MMP13 expression widely dispersed into the growth plate proliferative zone (3) differences in growth plate maturation zone sizes (4) a dramatic acceleration of growth plate closure with bone bridge formation particularly clustered to medial areas where OA later predominates^35^. Together these studies suggest that STR/ort mice have an inherent endochondral ossification defect that drives their OA pathology. Interestingly, the relationship between longitudinal long bone growth rates and OA development in humans is a completely unexplored area. It is intriguing nonetheless that canine hip dysplasia, a hereditary predisposition to degenerative OA, is more common in certain breeds, in particular those larger breeds which tend to grow more rapidly^74^.

## STR/ort mouse immune phenotype

Although OA is not primarily a classic inflammatory disorder, it is accepted that cytokines play an important pathogenic role^75^. Indeed, Chambers *et al.* (1997) found elevated IL-1β levels in AC chondrocytes of STR/ort mice at all ages^56^. In addition, serum levels of IL-1β, IL-4, IL-10, interferon γ were markedly higher in STR/ort mice^76^. Our previous microarray analysis in AC identified NFkB signalling as the main pathway modified in STR/ort mice (vs CBA^11^) and immunolabelling for the NFkB subunit p65 confirmed elevated levels in AC chondrocytes of STR/ort mice from 8 weeks of age^11^. The NFkB pathway is a recognised hub for inflammatory signalling which suggests links between chondrocyte cytokine production and signalling and catabolic changes in OA cartilage in STR/ort mice.

Together these studies suggest that STR/ort OA has an important inflammatory component and this is further cemented by observations of spleen and lymph nodes abnormalities^16^. We more recently showed that male STR/ort mice possess significantly bigger spleens (with greater cellularity), decreased naïve T cell numbers, but increased activated T and B cell numbers, indicating a heightened inflammatory status^11^. This could perhaps be explained by the high bone mass phenotype of STR/ort mice (described above) and compression of the bone marrow necessitating extramedullary hematopoiesis^70^. Oxidative stress is associated with increased inflammatory mediator production and as such, reported increases in oxidative stress in STR/ort mice (see above) may provide an alternative explanation for these raised inflammatory markers levels^63^. These studies suggest a central function of inflammatory pathways to in STR/ort mouse OA development; they may also reflect a common molecular aetiology linking these OA and immune phenotypes.

STR/ort mice exhibit increased AC expression of beta-defensins 3 and 4, broad-spectrum antimicrobial components of innate immunity. These findings offer a link between host defence mechanisms and inflammation with AC tissue-remodelling processes^77^. Moreover, it is recognised that CGRP may contribute to human joint pain and CGRP/CGRP receptor signalling may indeed be modified in STR/ort mouse synovium via increased CD11c(+) macrophages, with high IL-1β in F4/80(+) and high CGRP, CLR, and RAMP1 in the F4/80(−) cell fraction, which can be ameliorated upon macrophage depletion, suggesting that synovial macrophages and IL-1β production may be suitable therapeutic targets for treating OA pain^78^.

## Obesity/metabolic syndrome in the STR/ort mouse

Obesity is now a recognised OA risk factor and it has been reported that the parent STR/1N strain exhibits higher blood cholesterol and phospholipids compared to DBA/2JN and A/LN strains^10^. STR/ort mice have also been described as hypercholesterolemic and hyperlipidemic (raised cholesterol, high-density and low-density lipoprotein, triglyceride and insulin) without different glucose levels compared to C57Bl6/J and CBA/JN^79^. Regardless, it has been suggested that STR/ort mice should not be termed obese as their weight is significantly lower than ob/ob mice^80^. STR/ort mice also show low levels of serum adiponectin, a key player in glucose and lipid metabolism, which resembles human primary hypertriglyceridemic patients. Despite this, a reduction in body weight of STR/ort mice, using fenofibrate treatment, did not modify serum lipid composition nor OA severity^81^, suggesting that lipid metabolism anomalies were not the primary cause of spontaneous OA in STR/ort mice.

More recently, microarrays on STR/ort AC/subchondral bone described upregulation of 331 genes related to development and function of connective tissues, and 290 genes downregulated linked to lipid metabolism, in particular genes that were directly interacting with peroxisome proliferator-activated receptor (PPAR) alpha/PPARgamma^82^. While PPARalpha and PPARgamma mRNA levels themselves were not significantly altered, multiple PPAR pathway components were, leading the authors to conclude that decreased PPAR signalling contributes to OA progression in STR/ort mice by promoting osteoblastogenesis and enhanced bone formation.

## Concluding remarks

The STR/ort mouse is an excellent model of spontaneous osteoarthritis with disease pathology starting early in life and showing many similar characteristics akin to human primary OA. The phenotype of the STR/ort mouse is well characterised with pathology observed in the knee, elbow, ankle and temporomandibular joints of male mice; this highlights the sexual dimorphism in this strain, whereby females show higher bone mass and protection from reproducible AC degeneration. The highly defined and reproducible disease pathology of the STR/ort mouse has, to date, offered the unique opportunity to identify the pathological role that key determinants of the AC and subchondral bone phenotypes play in spontaneous OA development, highlighting the attractiveness of this murine model in exploring the aetiopathogenesis of spontaneous OA. Whether research in the OA field should focus upon pre-clinical studies or on clinical studies in man is still a matter of debate, and has been elegantly debated in a recent editorial by Hunter and Little^1^. However, with new acceptance that broad generalisation regarding OA aetiopathogenesis is somewhat distracting and flawed in our pursuit of a single disease-modifying treatment, research in the OA field will undoubtedly look to utilise animal models such as the STR/ort mouse to yield greater understanding of primary OA. The recent research discussed herein certainly indicates that a better understanding of the genes, molecules and processes contributing to STR/ort mouse OA will aide significantly in the identification of new preventative, protective and curative avenues for OA.

## Author contributions

All authors were fully involved in the preparation of this manuscript and approved the final version.

## Competing interest statement

The authors have no potential conflicts of interest to disclose.

## Funding

The authors wish to acknowledge Arthritis Research UK for funding (20413, 20581, 20258 and 20859).
